# Usability and acceptability of ambulatory monitoring in undiagnosed syncope: insights from the ASPIRED-Q qualitative study

**DOI:** 10.1136/bmjopen-2024-095927

**Published:** 2025-04-08

**Authors:** Alice Pearsons, Coral L Hanson, Lis Neubeck, Caroline Blackstock, Ellise Clarke, Matthew James Reed

**Affiliations:** 1 Centre for Cardiovascular Health, Edinburgh Napier University, School of Health and Social Care, Edinburgh, UK; 2 Emergency Medicine Research Group Edinburgh (EMERGE), Royal Infirmary of Edinburgh, NHS Lothian, Edinburgh, UK; 3 Acute Care Edinburgh (ACE), Centre for Population Health Sciences, Usher Institute, University of Edinburgh, Edinburgh, UK

**Keywords:** ACCIDENT & EMERGENCY MEDICINE, eHealth, Cardiology

## Abstract

**Abstract:**

**Objectives:**

The aim of this study, which was embedded into the ASPIRED randomised controlled trial (ISRCTN10278811), was to explore patient and healthcare professional usability and acceptability of an enhanced (14-day) ambulatory ECG monitoring patch to manage and facilitate discharge of emergency department patients with unexplained syncope.

**Design:**

A qualitative study using semistructured interviews. Data were analysed using thematic analysis and mapped using the theoretical framework of acceptability.

**Participants:**

A sample of 20 syncope patients recruited to the ASPIRED RCT and 10 healthcare professionals who had a direct care provider and clinical decision role for syncope patients (eg, consultants, junior doctors, advanced nurse practitioners, advanced care practitioners, emergency nurse practitioners and physician associates) were recruited from four hospitals (two in England and two in Scotland) between February 2023 and January 2024.

**Results:**

Three overarching themes developed that mapped to six of the seven constructs within the theoretical framework of acceptability. The themes were: (1) Efficacy: Patients and healthcare professionals felt that the remote intervention would increase patient reassurance. Healthcare professionals perceived the intervention would improve clinical care pathways by overcoming delays for Holter monitors, but that a standard protocol would be required to ensure appropriate intervention use. (2) Burden: Patients considered that the device was non-obstructive and easy to use. However, healthcare professionals noted that although attaching the device was simple, there would be associated time and resource costs (eg, documentation). (3) Communication and education: Comprehensive verbal and written information were considered necessary to ensure that the intervention was usable by, and acceptable to, patients. Healthcare professionals suggested additional training would be required. Additionally, they considered that feedback from patient monitoring would reinforce their decision-making and improve healthcare professionals’ self-efficacy to use the device appropriately.

**Conclusions:**

An immediate, enhanced (14-day) ambulatory ECG monitoring patch was positively received by patients and offered healthcare professionals an acceptable route for monitoring emergency department patients with unexplained syncope. However, future use should be controlled using standardised pathways to prevent inappropriate use.

**Trial registration number:**

ISRCTN10278811.

**STRENGTHS AND LIMITATIONS OF THIS STUDY**

- A qualitative methodology enabled in-depth exploration of patient and healthcare professional perspectives.
- This study recruited from four large tertiary hospitals from across the UK, two in Scotland and two in England.
- The use of the theoretical framework of acceptability provided a structured analysis of themes enhancing the robustness of the findings.
- This study experienced challenges following up patient participants to interview after initial consent.
- The majority of patients (60%) had only experienced one syncope episode at the time of the interview; therefore, it may not be possible to generalise these findings to populations who experience recurrent undiagnosed syncope.

## Introduction

 Syncope is a clinical syndrome associated with transient loss of consciousness and accounts for approximately 1%–2% of emergency department (ED) presentations.^1 2^ Healthcare professionals (HCPs) are required to diagnose and risk stratify this population to identify patients at higher risk of cardiovascular events or death.^3^ This can be complicated as syncope involves spontaneous and complete recovery; therefore, initial examination and clinical investigations may be normal. Testing has a low diagnostic yield that often fails to identify underlying aetiology.^4^ This has resulted in admission to hospital rates for syncope in Europe of between 27% and 56.6%,^5^,^7^ contributing to significant healthcare costs, and 36.6% of patients still being discharged without a diagnosis.^8^ Those with unexplained syncope are often referred for additional tests including advanced imaging and are at increased risk of readmission. Recurrent unexplained syncope increases the risk of injury and anxiety, and the loss of permission to drive, all of which increase the potential for disruption in family and role functions.^9^

The unpredictable and episodic characteristics of syncope necessitate the need for efficient and timely diagnostic tools. External prolonged ECG monitoring is often undertaken in those with unexplained syncope and is recommended when arrhythmic syncope is suspected.^10^ Commonly used monitoring technologies include Holter monitors and implantable loop recorders (ILRs). However, access to these services is subject to delays. The average waiting time to receive a Holter monitor ranges between 5 and 12 weeks.^10^,^12^ There is increasing evidence that the majority of arrhythmic outcomes experienced by medium and high-risk patients can be identified within 15 days of the index ED syncope presentation,^13^,^15^ and monitoring should, therefore, be initiated as close to the index visit as possible. To address these challenges, innovative solutions are being explored.

Novel ambulatory cardiac monitors are non-invasive, water-resistant, have no leads or wires^16^ and are CE-marked for clinical use in the UK. The ASPIRED randomised controlled trial^17^ is the first large study to look at applying a device immediately after the presenting syncope episode (ie, from the ED) and monitoring for an enhanced period (ie, 2 weeks). The study recruited 2234 adults 16 years or older presenting with syncope remaining unexplained after initial assessment, with a primary objective to determine whether the intervention decreases the number of self-reported episodes of syncope at 1 year compared with standard care monitoring. The study will also determine whether the strategy increases detection and diagnosis compared with standard practice. ASPIRED hypothesised that applying continuous cardiac monitoring early after syncope at the index visit would be the optimum strategy to detect, diagnose, treat and exclude underlying cardiac arrhythmia in patients with unexplained syncope. The control arm involved patients receiving all care usually given to unexplained syncope patients at each participating site along with some form of standard local monitoring such as but not limited to wired inpatient telemetry, Holter style monitoring or ILR (online supplemental file 1). The potential benefits of novel prolonged monitoring devices extend beyond just timely diagnosis. These devices are designed to improve patient comfort and compliance due to their user-friendly features, which may lead to better patient adherence to continued wearing of the monitoring and subsequently higher diagnostic yields.^16^ The aim of the current study was to explore the usability and acceptability of the ambulatory monitoring intervention for patients and HCPs. These are crucial for the implementation of new technologies in clinical practice. Understanding the perceptions and experiences of patients and HCPs can inform the development of effective strategies to integrate such devices into routine care, potentially transforming the management of unexplained syncope and reducing associated healthcare burdens.

## Methods

This qualitative study collected data via individual semistructured telephone interviews. The reporting of the study followed the Consolidated criteria for Reporting Qualitative research (online supplemental file 2).^18^

### Participants

This study recruited patients and HCPs from four UK NHS EDs (two in Scotland and two in England), which were recruiting to the multicentre open-label randomised controlled trial of immediate enhanced ambulatory ECG monitoring versus standard monitoring in acute unexplained syncope patients (The British Heart Foundation funded ASPIRED study (https://www.isrctn.com/ISRCTN10278811).

Eligible patients were those with an ED attendance for syncope that remained unexplained after initial ED or acute medicine unit (AMU) assessment and who were recruited into either the intervention or control arm of the ASPIRED trial. Eligible ED or AMU HCPs were those who had a direct care provider and clinical decision role for syncope patients (eg, consultants, junior doctors, advanced nurse practitioners, advanced care practitioners, emergency nurse practitioners and physician associates). HCPs recruited were aware of the trial and device and may have helped identify patients for recruitment. However, they were not involved in the application or delivery of the intervention which was managed via research teams at each site.

The intention was to recruit 18 patients (12 from the intervention group and 6 from the control arm) and 12 HCPs. Taking part was voluntary and did not impact patients’ participation in the ASPIRED trial. All participants were recruited via onsite clinical research teams who provided study information and obtained in person written consent, which was returned via post to researchers at Edinburgh Napier University.

### Patient and public involvement

Our ASPIRED study patient and public involvement group is made up of patient representatives, lay members and a representative from the Arrhythmia Alliance. They have been involved in informing the ASPIRED and ASPIRED-Q study research questions, study protocol and the development of patient-facing information. They have continued to be involved throughout the study and dissemination processes.

### Data collection

Data collection took place between February 2023 and January 2024 using semistructured telephone interviews. Interviews were conducted by AP, an early career qualitative researcher with a master’s degree who works as a research fellow, with mentoring from CLH, a PhD qualified researcher with 10 years of qualitative research experience. AP was an experienced ED nurse who had no previous relationship with study participants. The interview guide consisted of open-ended questions (online supplemental file 3). It was developed and refined through discussion among syncope HCPs and Edinburgh Napier University researchers. For both the intervention and control patient groups, interviews were opened with broad questions on experience(s) of syncope, time in the ED and ongoing care postdischarge. For those who received the ambulatory monitoring intervention, questions focused on user experience of the device and how it may or may not have impacted on their care. HCP interviews focused on syncope management in the ED before delving into the challenges of syncope care with no known cause and how having access to an ambulatory prolonged monitoring device may impact on their decision-making process for this cohort of patients. All interviews were audio recorded and transcribed verbatim using an external transcription service. Field notes were taken throughout the interviews to capture context, quality of the interaction and reflections on researcher bias.

### Data analysis

Data were analysed using the five-stage framework approach^19^: stage 1 familiarisation with data; stage 2 construction of a thematic framework; stage 3 indexing and coding; stage 4 data summary and display and stage 5 mapping and interpretation.

Transcripts were reviewed by one researcher (AP) to ensure accuracy of the transcript, increase familiarity with the data and to remove any identifiable information. This included listening back to participant interview recordings. Inductive open coding was initially undertaken by AP for 10 transcripts (five patient transcripts and five HCP transcripts), CLH independently coded six of these transcripts to minimise bias and increase reliability. Coding was undertaken using NVivo V.14 (QSE International, Melbourne, Australia) and 92 initial open codes were generated. Discussions between AP and CLH refined the initial open codes into seven initial themes: experience of syncope, acute care journey, acceptability and usability of the intervention, decision-making, communication, resources and impact on life. These seven initial themes were considered as a working analytical framework to guide further analysis. Following this, one researcher (AP) applied this analytical framework across all transcripts. During the entire process of analysis, discrepancies in the classification of codes and categories were resolved between authors (AP, CLH, LN and MJR).

This process highlighted the need to apply a theoretical framework of acceptability to the developing themes. Specifically, the initial themes highlighted a complex interaction between patients’ understanding of the intervention, its perceived usability and the broader context of their medical experiences. As we refined the analysis, it became evident that these experiences were multifaceted and not just related to practical concerns (such as the usability of the intervention) but were also deeply intertwined with what participants deemed acceptable or unacceptable in terms of their treatment and decision-making processes. Therefore, the theoretical framework of acceptability^20^ was chosen to ground analysis in theory regarding the acceptability of an intervention from both an intervention deliverer and recipient perspective with a prospective and retrospective lens. By doing so, it is possible to assess the anticipated and experienced acceptability of the intervention. The seven-component framework includes affective attitude, burden, perceived effectiveness, ethicality, intervention coherence, opportunity costs and self-efficacy.^20^ Therefore, the model was used to increase the robustness of analysis by applying the initial themes against predefined components for acceptability, which increased coherence and consistency of interpretation within the systematic framework. This helped refine the initial themes into main and subthemes to aid understanding.

## Results

**Figure 1:**
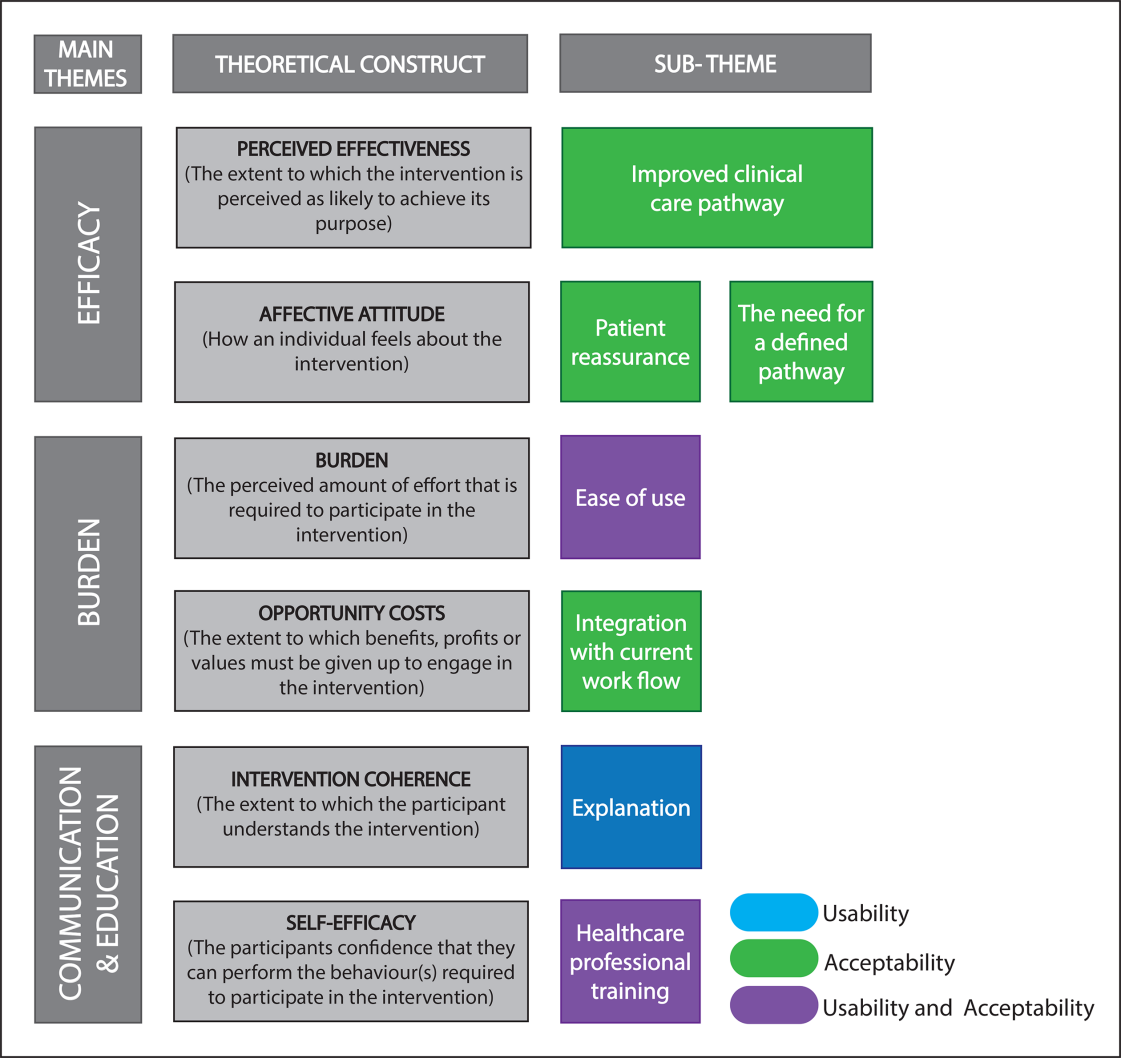
Overarching themes mapped with the theoretical framework of acceptability.^20^

43 participants were recruited from 4 hospitals (2 in England and 2 in Scotland). Of these, 30 (69.8%) completed interviews (20 patients, 10 HCPs), 12 (27.9%) could not be contacted to arrange the interview and 1 (2.3%) declined to participate once contacted. Most patient participants were male (n=13, 65%), median age 66 years (IQR 17) and 60% (n=12) experienced only a single syncope episode. One patient participant reported more than five syncope events. HCPs were made up of 2 ED consultants (20%), 5 ED specialty registrars (50%), 1 acute medicine specialty registrar (10%) and 2 physician associates (20%). Interviews ranged from 8 to 36 min (patients) and 36 to 57 min (HCPs). Usability and acceptability were mapped within the theoretical framework of acceptability^20^ (figure 1). Indicative quotes are presented in tables at the end of each section.

### Overarching themes

The thematic analysis resulted in three overarching themes (efficacy, burden, and communication and education) and seven subthemes that mapped to six of the seven constructs within the theoretical framework of acceptability (figure 1).

### Main theme 1: efficacy

The theme of efficacy reflects patient and HCP participant perceptions of how effective the intervention was at improving syncope management, influencing confidence in the intervention, and ultimately the overall experience and acceptability of the intervention. This theme incorporates the constructs perceived effectiveness and affective attitude from the theoretical framework of acceptability (figure 1). Perceived effectiveness encompasses the subtheme improved clinical care pathway and affective attitude encompasses two subthemes (1) patient reassurance and (2) the need for a defined pathway. Indicative quotes for subthemes are presented in table 1.

**Table 1:** Quote supporting efficacy theme

Theoretical construct	Subtheme	Quotes
Perceived effectiveness	Improved clinical care pathway	“I mean, a lot of the time, the GPs say, you know, go to your GP and get a tape, like I’m just, sort of, sending them off into the ether and hoping that they either have the initiative to book and feel that it’s important enough to follow up, or that their GP has enough availability for them to make sure that happens. So, it’s better for continuity of care in that if we’re able to organise that onsite.” (HCP 10)“Also, it shows the patients that we are taking it seriously, and this isn’t scientific, but it feels that it just has to be better than them waiting four months to get a 24-hour tape.” (HCP 5)
Affective attitude	Patient reassurance	“… I think sometimes, if we can discharge this set of patients, having that bit of reassurance I think would be good for us and also the patient.” (HCP 8)“I think it’s really good, I think that it’s definitely helped [intervention] patients feel reassured as well as provides a good way to investigate a collapse that we’ve been worried about further as an outpatient…” (HCP 6)“I was a bit scared because, obviously, I was scared it was going to happen again. And then idea of something could be wrong but it wasn’t being monitored, I was a bit scared about that” (Participant 11, control group)“And in a sense, it could be said that the only thing that had brought it into my mind that I might have this fainting fit again was the fact that the device that I was given was set up to deal with the fact that this might happen again. So, I think that brought the idea of the possibility of it happening again into my mind where it wouldn’t have been there otherwise.” (Participant 12, intervention group)
	The need for a defined pathway	“if these bodyguard mini, kind of, monitors do become routine practice we will definitely need a standard operating procedure for how to deal with the resets…outside the context of a trial, I think a new SOP for how these a followed up would definitely be needed.” (HCP 5)“… it would probably end up being overused, because actually, as well as it being a useful to get home patients who are on the … it might be that it’s then just used as, like, a, oh, I may as well do this even though I’m not worried about this patient, type of thing. And actually, you end up with somebody who’s quite clearly fine, getting on… I think it would be overused when it initially, if it initially ever came into be in rules.” (HCP 7)

HCPhealthcare professional

### Improved clinical care pathway

The management of syncope was frequently described by HCPs as akin to solving a puzzle. Despite universal awareness and utilisation of syncope guidelines or pathways to inform clinical decisions, HCP participants noted that clinical decisions, particularly regarding patient admission or discharge, were not always straightforward. They believed that applying an ambulatory prolonged monitoring device prior to discharge could significantly enhance clinical decision-making, continuity of care and patient healthcare experience, and expedite follow-up monitoring where necessary. Such a device was suggested to optimise the discharge process for patients categorised as intermediate risk, potentially reducing unnecessary admissions without compromising continuity of care. Anecdotal evidence from HCP participants indicated that offering ambulatory monitoring options during an ED visit could mitigate the risk of patients not receiving recommended monitoring postdischarge. Overall, the ambulatory prolonged monitoring device was thought to improve patient healthcare experience by providing better treatment than standard care.

### Patient reassurance

Patient participants in the control and intervention group expressed a range of emotions related to their syncope and subsequent presentation to the ED with predominant feelings of worry about the severity of the underlying cause and potential for future incidents. There was a sense of frustration over the lack of definitive diagnosis when numerous tests returned normal results. The frustration was alleviated when HCPs effectively communicated that, although a definitive cause was not identified, serious conditions had been ruled out at that time. Those in the control group were left with residual fear at discharge about repeat events in the future due to delays or complete lack of ongoing monitoring, as well as a feeling that there was not any concern on the HCPs’ part regarding their situation. This fear was not as pronounced in the intervention group as there was a feeling that they would receive ‘quicker’ help than waiting for a general practitioner. Despite this, one intervention patient participant felt that wearing the device was the reason he continued to think about the possibility of repeat events, suggesting that without this, they would not have had these ongoing concerns.

HCPs were aware that patients experienced anxiety on discharge from the ED following an unexplained syncope. The anxiety was exacerbated by complex relationships with primary care providers and lengthy delays in accessing Holter monitoring and follow-up tests. Therefore, HCP participants believed that the ability to offer patients immediate ambulatory monitoring would be positively received and serve to provide patient reassurance. Patient and HCP participants felt that the provision of immediate monitoring before discharge showed patients that HCPs were taking their condition seriously and that any future events would be captured, facilitating diagnosis and potentially preventing serious future incidents.

### The need for a defined pathway

Despite highlighting the benefits of an ambulatory monitoring intervention, HCP participants expressed concerns about the future workflow if the intervention were implemented on a larger scale. A primary concern was that the success of the intervention could lead to resource limitations, potentially hindering its efficacy. HCP participants noted the possibility of inappropriate usage, where the availability of the device might encourage its use on low-risk patients who do not necessitate such monitoring. Therefore, to ensure sustainable implementation within routine practice, they identified the need for a clear pathway or standard operating procedure. This would help delineate appropriate usage criteria and maintain resource allocation, ensuring that the intervention remains effective and sustainable.

### Main theme 2: burden

The theme of burden in patient and HCP participant perceptions of the physical, emotional and logistical challenges associated with the intervention. This main theme incorporates the constructs of burden and opportunity costs from the theoretical framework of acceptability (figure 1). With the subthemes: (1) ease of use, (2) integration with current workflow and (3) future resource concerns. Indicative quotes for subthemes are presented in table 2.

**Table 2:** Quotes supporting burden theme

Theoretical construct	Subtheme	Quotes
Burden	Ease of use	“…you more or less forget about it, once you’ve got it put on. It was really handy, actually” (Participant 23, intervention group)“No disadvantages whatsoever. Because I could do everything that I'd normally do, with’ the monitor on.” (Participant 21, intervention group)“[went swimming] twice a day, roughly one hour… I’m trying to think…the one time it was itchy, and I don't think it was the swimming, I’ve no idea why.” (Participant 29, intervention group)“I just really found it awkward to switch on… And you couldn’t really tell whether it was on or not. There was a little light that flashed, but it flashed that quick that you could…hardly see it. You had to put your hand over it…so you could see if it was flashing or not.” (Participant 23, intervention group)“It can get in the way a bit, I don’t know, when you’re getting dressed, it can get caught on your clothes and it’s a bit uncomfortable to sleep with it on because it digs into your skin.” (Participant 18, intervention group)“…actually forgot sometimes it was there, I had to go and scratch my chest or whatever to feel this thing. And it wasn’t…no one commented on it so I didn’t feel conscious about it or anything like that.” (Participant 25, intervention group)“I had to wear a higher neck top to hide it. Once it was on after a couple of days, I just became used to it”. (Participant 19, intervention group)
	Technical issues	“After about ten days, it started going off in the middle of the night. I’m thinking, what’s going on? I didn’t realise that was the noise that would come out of it. It had come unstuck, it lost contact, and so I re-attached it. Then after about 13 days, the battery actually failed, it just ran out of battery.” (Participant 20, intervention group)“I had a couple of times where it seemed to have lost contact, which wasn’t very good but then it fixed itself, and I think sometimes that happened when I was sleeping because I lie on my front when I sleep.” (Participant 25, intervention group)
Opportunity costs	Integration with current workflow	“I guess, because it’s a relatively quick and easy thing to do it would work very well if we can do that in the emergency department and it saves the patient coming back and it saves someone else’s time to apply i…t’ (HCP 6)‘Yes. And not only that, the documentation and all of it, it is not just that…[the] procedure as such doesn’t take that much, but then there are a lot of other things around…getting the monitor, getting the equipment. So, it is not just the attaching time; it’s the other things around it which takes a while” (HCP 9)
		“Like if you’re going to give an iron injection how many seconds would that take? It would take less than ten seconds. But to get the medication, to get the syringe, to get the needle, so that takes more. And then to document that you have given the iron injection. It’s the same with this monitoring…” (HCP 9)

HCPhealthcare professional

### Ease of use

Patient participants who received the monitor reported that it was generally easy to wear, though some found it sat too high on their neck, making it quite visible and, in one case, awkward. However, for most people, the device was unobtrusive and did not disrupt their daily activities. One patient participant even continued their daily swimming routine. Some female patients reported being slightly more aware of the device and had to alter their choice of clothing to conceal it. Over time, patient participants often forgot the device was attached.

While most patient participants had no issues using the device, several reported that their batteries failed, and they were not provided with a charging cable or instructions on how to charge it. Some were incorrectly informed that the battery was fully charged and would not need recharging, only for it to fail before the end of the 2-week monitoring period. Additionally, patient participants were sometimes confused by what colour the flashing lights on the device were or seeing the lights themselves due to the device’s position or poorer eyesight. Some reported loud noises coming from the device which they had not been warned about. In most cases, this was due to lost contact by the electrode, which for one person resulted in them being woken up in the middle of the night. Despite any discomfort or inconvenience, patient acceptability of the intervention remained high. All intervention patients regardless of personal experience, for example, whether they were injured during their syncope, were working, had other health issues or varying hobbies reported they wore the device for the entire 14 days, unless the battery failed. This displays a very high level of acceptability among a very diverse patient group.

### Integration with current workflow

HCP participants considered how the current intervention would fit into their current workflow. They believed that attaching and explaining the device as part of the syncope discharge conversation would be feasible, as the time taken to attach the device and discuss its use would be equivalent to providing discharge medications and advice. However, HCP participants highlighted it was the tasks associated with the device, such as documentation and locating the device and chargers in the department, that could be more resource intensive. Despite these concerns, the idea of implementing an ambulatory prolonged monitoring device within the ED was positively received and believed to integrate well into current syncope management practices.

### Main theme 3: communication and education

The theme of communication reflects patient and HCP participant perceptions of the clarity and effectiveness of information provided by HCPs which directly influenced patient sense of acceptability and engagement. The main theme incorporates the constructs intervention coherence and self-efficacy from the theoretical framework of acceptability (figure 1) with the subthemes (1) explanation and (2) HCP training. Indicative quotes for subthemes are presented in table 3.

**Table 3:** Quotes supporting communication and education theme

Theoretical construct	Subtheme	Quotes
Intervention coherence	Explanation	“When I was discharged from the hospital was I given any particular advice? No… she said, there wasn’t anything. They just simply said I was okay, fit to go home now.” (Participant 13, control group)“They were going to keep me in for three days because I heard them outside the curtain in the hospital talking to the consultant…[but] there [were] no beds” (Participant 14, control group)“…the booklet …it’s not clear. And I put it… then a couple of days later [the device] started making funny flashing and noises. So… I went to see a geeky friend of mine with this booklet. And he read through it, because I hadn't been given any charger or anything. So, there’s this booklet…but it says in one of the solutions, if it persists, replace electrode. Now, do you know what to do to replace electrode? It’s not the battery.” (Participant 27, intervention group)“it was, the instructions in the booklet are pretty clear and the woman on the phone I was speaking to at the time, she also…I think she had the booklet in front of her as well, she was walking through and elaborating on bits where she may be felt was confusing. But, no, it was easy enough, you take the sticker off the back, stick it on your chest, double check that the little green lights appearing and away you go.” (Participant 25, intervention group)
Self-efficacy	Healthcare professional training	“If I was talking them through the device prior to discharge, I feel like it would be no different than talking them through their discharge meds. As long as you have adequate training, and I’m confident in explaining how to put it on and how it works et cetera, then it’s just part of your discharge chat” (HCP 8)“Because especially after I had the feedback that this [patient] in the study and he had something significant, you actually feel more empowered that you should be doing this and it’s actually helping and working. You can see a real tangible result out there. Really appreciate the feedback. Even if it’s a feedback that, oh, we did a 14 day, found nothing…But when you get a positive impact, you feel much better and you feel that okay, yeah, things are working. You were thinking in the right direction” (HCP 1)

HCPhealthcare professional

### Explanation

Most patient participants in the control and intervention group reported a lack of involvement in the ED regarding the tests undertaken, care plan discussions and discharge decisions. One patient participant overheard they were getting discharged from behind their cubicle curtain when HCPs identified that there were no beds for admission. This lack of involvement was further felt by a perceived lack of discharge advice. This was contradicted by HCPs, where all HCP participants reported that they provided comprehensive discharge advice with a focus on red flag warnings and clearly communicating that they were unable to find a cause but could rule out anything serious. For patient participants, the feeling of lack of involvement was exacerbated in the control group who did not benefit from the additional syncope advice experienced as part of the device instruction set up, which included a leaflet on syncope.

All intervention patients reported receiving verbal instruction on the device. For some, this was in person prior to discharge from the ED, while for others this was over the telephone after the device was posted out to their home address. Regardless of the mode of communication, intervention participants generally felt sufficiently verbally informed, and no obvious differences in terms of acceptability were noted between the methods of information delivery. However, written information in the form of a leaflet provided with the device was highly valued. It was felt that this reinforced the information that was provided at the time of recruitment when patients may not be able to easily digest and remember verbal information. Some patient participants found the manual contained complex terminology, which hindered their ability to self-manage the device or troubleshoot issues when they arose.

### HCP training

HCP participants exhibited a willingness to embrace the long-term ambulatory monitoring strategy to manage unexplained syncope in the ED. However, they recognised that additional training would be necessary to appropriately attach the device and counsel patients, should this intervention become part of routine practice. Effective training programmes would ensure that HCPs are confident and competent in using the device, which is essential for its successful implementation and integration into standard care protocols. Two HCP participants reported that receiving feedback from patients’ monitoring periods reinforced their decision-making processes and enhanced their self-efficacy in using the device appropriately. This feedback loop was considered crucial as it not only validated HCPs’ actions but also fostered continuous learning and improvement in patient care.

## Discussion

We analysed patient and HCP views about the implementation of a novel ambulatory prolonged cardiac monitoring device designed to increase detection and diagnoses of unexplained syncope. HCP and patient participants considered that the benefits of such a device were its capacity to improve clinical decision-making and to reassure patients. The device was considered easy to use by patient participants, despite some reported technical issues. HCP participants reported that it could be integrated into their workflow but that there was a need for a defined pathway to ensure appropriate use. HCP and patient participants considered that improved written information about device use and clinician training were necessary to ensure that the device could be successfully implemented in routine practice.

Compared with Holter monitors, patch monitors offer several key advantages: extended monitoring periods,^21^ improved patient comfort and compliance,^22^ lower risk of artefact and interruption,^23^ simpler use,^24^ higher diagnostic yield^25 26^ and lower cost per diagnosis.^27^ A previous systematic review of wearable vital sign monitoring reports mixed patient experiences including physical discomfort of the device, concerns about reliability and apprehension that using the device would reduce engagement with nurses.^28^ These concerns were not highlighted within our study. Instead, patients reported increased reassurance and a stronger sense of involvement in their care. This was true regardless of how the patient presented to the ED, for example, number of previous syncope or whether they experienced injury suggesting the devices’ appropriateness for this population. The technology acceptance model^29^ suggests two main factors that determine an individual’s acceptance of technology: perceived usefulness and perceived ease of use. Patients are more likely to accept a technology if they believe it will improve their healthcare experience and are given realistic expectations of the technology capabilities,^30^ in this current study, this related to perceived quicker access to monitoring on discharge. Despite patient and HCP participants reporting high levels of acceptability, consideration still needs to be given to future intervention sustainability.

Ensuring intervention sustainability outside of clinical trial environments is an ongoing challenge. A key enabler to ensure sustainability is clear accountability of roles and responsibilities.^31^ In this study, HCP participants were uncertain about who should take responsibility for the intervention. In ASPIRED, 14-day ambulatory ECG results were shared with the participant’s responsible treating clinicians to action (usually the inpatient specialty physician and any relevant outpatient physicians responsible for ongoing care). ECG results were also placed in the participant’s health record to be available to future treating clinicians. It was thought feasible to attach the device at discharge, reportedly taking no more time than a discussion about discharge medication; however, this perception was anecdotal, as most HCPs had not personally engaged in the process. There is concern that cumulative use of information technologies in healthcare contributes to burn out.^32 33^ What appears on its own to be a simple additional task can accumulate with other small tasks, becoming an occupational stressor.^34^ HCPs also need the right skills, knowledge and training to use technologies competently within clinical guidelines.^35^ HCP participants in this study suggested that successful integration into the current workflow would require the patch monitor to be written into existing syncope pathways. However, 55% of HCPs across Europe either lack or do not adhere to specific protocols for syncope evaluation.^36^ Therefore, variability in practice may present challenges for the widespread adoption of ambulatory patch monitors. Future research should focus on how to best incorporate this intervention into existing workflows and determine which workforce should manage this task.

### Strengths and limitations

The strength of this study includes its recruitment from four large tertiary hospitals from across the UK. This allowed for in-depth exploration of patient and HCP perspectives. The use of the theoretical framework of acceptability provided a structured analysis of themes enhancing the robustness of the findings. Limitations include the difficulty in engaging patients in the interviews. This may have introduced bias by perhaps interviewing patients who were more satisfied with their care or had more positive experiences; therefore, those who had negative experiences or felt the intervention was ineffective might have been under-represented. Additionally, 60% of those who took part had a single episode of syncope at the time of interview; therefore, it may not be possible to generalise these findings to populations who experience recurrent undiagnosed syncope. It is unlikely that data saturation was reached within the HCP participant group. This was due to challenges engaging this group, in particular recruiting nursing professionals, for example, emergency nurse practitioners. It is hypothesised by the authors that this relates to the lack of admin time provided to this professional group. Therefore, the findings of the HCPs should be interpreted with caution as they may not be representative of the ED as a whole.

## Conclusions

Novel ambulatory prolonged cardiac monitoring in an ED setting was positively received by patients and HCPs to manage unexplained syncope. This study suggests that successful implementation requires addressing the practical challenges of resources, communication and education. However, it offers significant opportunity to enhance efficiency of syncope management practices and importantly improve patient-centred care within an ED setting.

## supplementary material

10.1136/bmjopen-2024-095927 online supplemental file 1

10.1136/bmjopen-2024-095927 online supplemental file 2

10.1136/bmjopen-2024-095927 online supplemental file 3

## Data Availability

Data are available on reasonable request.
