# Supplementary figures and images for: Usability and acceptability of ambulatory monitoring in undiagnosed syncope: insights from the ASPIRED-Q qualitative study
Source: BMJ Open. 2025 Apr 8;15(4):e095927. doi: 10.1136/bmjopen-2024-095927 (PMC11979494; doi:10.1136/bmjopen-2024-095927)

## Supplementary file 1: Schematic diagram of ASPIRED RCT

<https://www.isrctn.com/ISRCTN10278811>

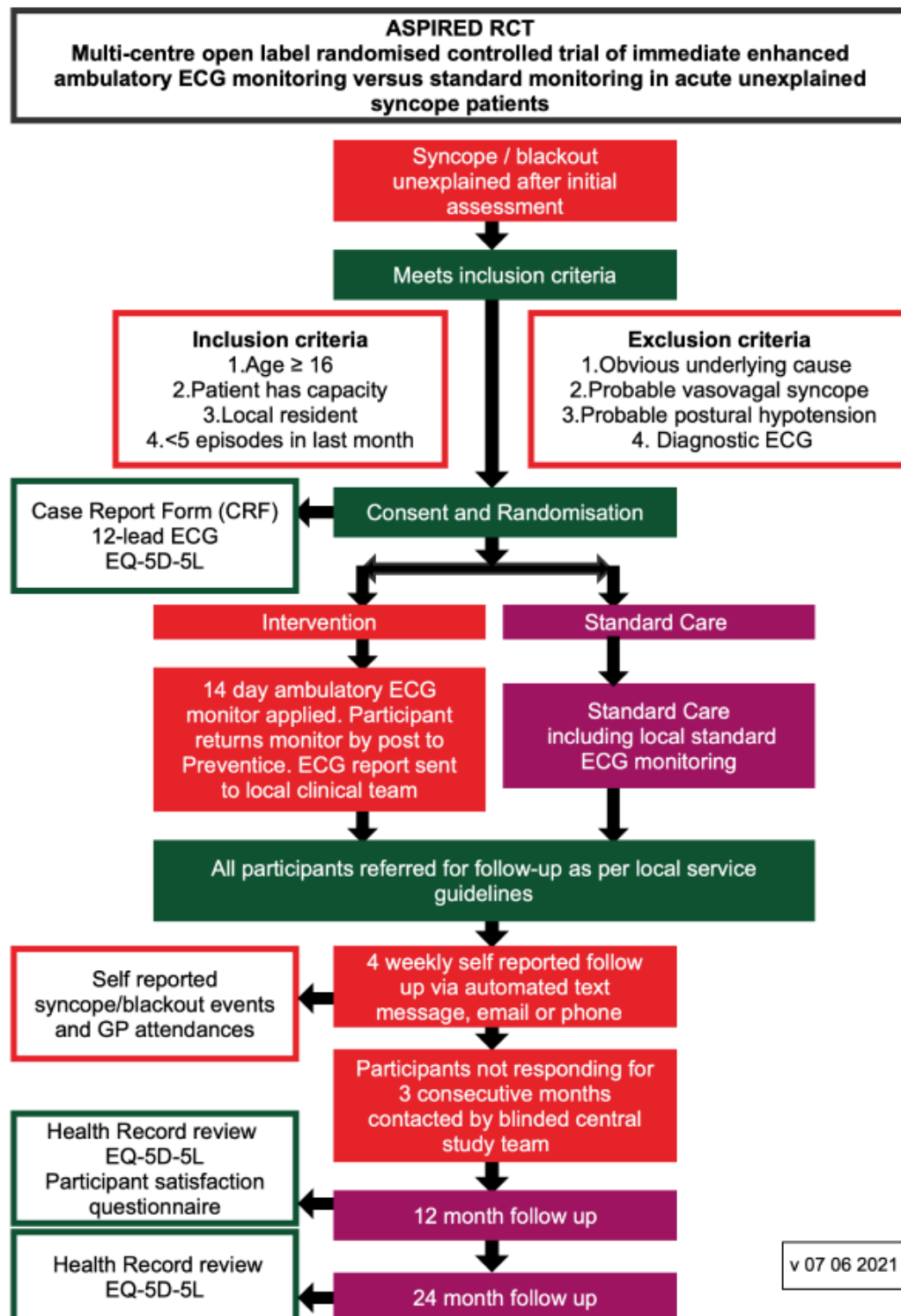

Supplement: online supplemental file 1 [file bmjopen-15-4-s001.pdf]
