# Supplementary material for: Usability and acceptability of ambulatory monitoring in undiagnosed syncope: insights from the ASPIRED-Q qualitative study
Source: BMJ Open. 2025 Apr 8;15(4):e095927. doi: 10.1136/bmjopen-2024-095927 (PMC11979494; doi:10.1136/bmjopen-2024-095927)
Supplement: online supplemental file 2 [file bmjopen-15-4-s002.pdf]

COREQ (COnsolidated criteria for REporting Qualitative research) Checklist

A checklist of items that should be included in reports of qualitative research. You must report the page number in your manuscript where you consider each of the items listed in this checklist. If you have not included this information, either revise your manuscript accordingly before submitting or note N/A.

**Topic**	**Item No.**	**Guide Questions/Description**	**Reported on Page No.**
**Domain 1: Research team and reflexivity**			
Personal characteristics			
Interviewer/facilitator	1	Which author/s conducted the interview or focus group?	AP data collection methods section (Page 3)
Credentials	2	What were the researcher's credentials? E.g. PhD, MD	AP has an MSc and is currently undertaking a PhD. All other authors had PhDs (Page 16)
Occupation	3	What was their occupation at the time of the study?	University based researchers (Page 6), and emergency medicine practitioners (page 16)
Gender	4	Was the researcher male or female?	First author (AP) female, other authors have not disclosed
Experience and training	5	What experience or training did the researcher have?	The first author (AP) has experience of qualitative studies as part of their PHD and has attended training on realist qualitative methodology. CLH is an experienced qualitative researcher with over 10 years' experience and closely mentored AP
Relationship with participants			
Relationship established	6	Was a relationship established prior to study commencement?	Participants were recruited via local hospital based clinical research teams. Therefore, there was no prior relationship at time of interview (Page 3)
Participant knowledge of the interviewer	7	What did the participants know about the researcher? e.g. personal goals, reasons for doing the research	Participants were aware that this was part of the larger ASPIRED clinical trial as part of their study information pack (not reported in manuscript).
Interviewer characteristics	8	What characteristics were reported about the inter viewer/facilitator? e.g. Bias, assumptions, reasons and interests in the research topic	Researchers kept field notes and reflective diaries to record contextual information about interaction quality and potential biases. Data collection section (Page 4)
**Domain 2: Study design**			
Theoretical framework			
Methodological orientation and Theory	9	What methodological orientation was stated to underpin the study? e.g. grounded theory, discourse analysis, ethnography, phenomenology, content analysis	Data were analysed via thematic analysis using the framework approach. Analysis section (Page 4)

			The theoretical framework of acceptability was used to structure analysis to assess the anticipated and experienced acceptability of the intervention (Page 5)
Participant selection			
Sampling	10	How were participants selected? e.g. purposive, convenience, consecutive, snowball	Participants were recruited alongside recruitment into a multi-centre open label randomised controlled trial. Participants section (Page 3)
Method of approach	11	How were participants approached? e.g. face-to-face, telephone, mail, email	Face to face via local clinical research teams. Participants section (Page 5-6)
Sample size	12	How many participants were in the study?	30 Results section (Page 9)
Non-participation	13	How many people refused to participate or dropped out? Reasons?	Forty-three participants were recruited from four hospitals (). Of these, 30 (69.8%) completed to interview (20 patients, 10 HCPs), 12 (27.9%) could not be contacted to arrange the interview and 1 (2.3%) declined to participate once contacted. Results section (Page 5)
Setting			
Setting of data collection	14	Where was the data collected? e.g. home, clinic, workplace	Data were collected by one researcher (AP) via individual semi-structured interviews using telephone. All interviews were audio recorded and transcribed verbatim using an external transcription service. Data collection section (Page 4)
Presence of non-participants	15	Was anyone else present besides the participants and researchers?	Not reported, but no others were present.
Description of sample	16	What are the important characteristics of the sample? e.g. demographic data, date	Data collection took place between February 2023 and January 2024 using semi-structured telephone interviews. Data collection section (Page 3)<br>Most patient participants were male (n=13, 65.6%).<br><br>The largest healthcare group was ED registrars (n=34, 46.6%), gender data were not collected for HCPS. Results Section (Page 5)
Data collection			
Interview guide	17	Were questions, prompts, guides provided by the authors? Was it pilot tested?	Interview guides provided (supplementary file 1)
Repeat interviews	18	Were repeat inter views carried out? If yes, how many?	Not applicable to study design

Audio/visual recording	19	Did the research use audio or visual recording to collect the data?	This study used semi-structured telephone interviews. Data collection section (Page 3)
Field notes	20	Were field notes made during and/or after the interview or focus group?	Researchers kept field notes and reflective diaries to record contextual information about interaction quality and potential biases. Data collection section (Page 4)
Duration	21	What was the duration of the inter views or focus group?	Interviews ranged from 8-57 minutes. Results section (Page 5).
Data saturation	22	Was data saturation discussed?	No
Transcripts returned	23	Were transcripts returned to participants for comment and/or correction	No
**Domain 3: analysis and findings**			
Data analysis			
Number of data coders	24	How many data coders coded the data?	Two, with feedback from all. Data analysis section (Page 5)
Description of the coding tree	25	Did authors provide a description of the coding tree?	Initial coding and reorganisation reported in Data analysis section (Page 4-5)<br>Figure 1 provides major themes and sub-themes (page 6)
Derivation of themes	26	Were themes identified in advance or derived from the data?	Initial themes were derived from the data and then classified using the theoretical framework of acceptability. Data analysis section (Page 5)
Software	27	What software, if applicable, was used to manage the data?	NVivo14, Data analysis section (Page 4)
Participant checking	28	Did participants provide feedback on the findings?	No
Reporting			
Quotations presented	29	Were participant quotations presented to illustrate the themes/findings? Was each quotation identified? e.g. participant number	Yes. (Tables I-III)
Data and findings consistent	30	Was there consistency between the data presented and the findings?	Yes – textual reporting reflected by the quotations. Results section (tables I-III)
Clarity of major themes	31	Were major themes clearly presented in the findings?	Yes – results reported by three overarching themes, tables, and Figure. Results section (tables I-III, Figure 1)
Clarity of minor themes	32	Is there a description of diverse cases or discussion of minor themes?	Yes results reported textual data. Results section (Page 5-13)

Developed from: Tong A, Sainsbury P, Craig J. Consolidated criteria for reporting qualitative research (COREQ): a 32-item checklist for interviews and focus groups. International Journal for Quality in Health Care. 2007. Volume 19, Number 6: pp. 349 – 357

Once you have completed this checklist, please save a copy and upload it as part of your submission. DO NOT include this checklist as part of the main manuscript document. It must be uploaded as a separate file.
