# Supplementary material for: Usability and acceptability of ambulatory monitoring in undiagnosed syncope: insights from the ASPIRED-Q qualitative study
Source: BMJ Open. 2025 Apr 8;15(4):e095927. doi: 10.1136/bmjopen-2024-095927 (PMC11979494; doi:10.1136/bmjopen-2024-095927)
Supplement: online supplemental file 3 [file bmjopen-15-4-s003.pdf]

Supplementary file 3: Patient Interview guide

Thank you for agreeing to talk to me about your care when you visited the Emergency Department because you lost consciousness (fainted or passed out). We are trying to better understand the patient experience of people like you so that we can improve the care that we deliver.

We will be recording this conversation, but it will remain completely anonymous. We understand that talking about any healthcare experience can be potentially distressing. You can skip to answer any question you like and end the interview at any time. Are you ok with that?

1) Tell me about your experience in the Emergency Department when you visited because you fainted or passed out.
2) What information did you get about your condition?
3) What treatment options did the doctor discuss with you?
4) What decisions were made about your medical care in the Emergency Department? (Probe about being admitted discharged)
5) How were these decisions made?
6) How did you feel about these decisions?
7) How involved did you feel in these decisions?
8) How much did you want to be involved in these decisions? (for example, about going home versus being admitted to the hospital?)
9) What information (if any) would you have wanted to receive that you did not get?
10) Have you had an episode where you lost consciousness previously? If so, I am going to ask you to complete a questionnaire about how this has impacted on your life
11) Is there anything you would like to add about losing consciousness and your care?

Intervention group only:
What advantages and disadvantages could you see from using the ambulatory ECG device that the ASPIRED trial is studying?

Staff Interview guide

1) Please tell me briefly about your general approach to the management of adult syncope patients in the ED.
2) How do you decide whether to admit, discharge, send to the observation unit, or send to outpatient follow up?
3) How much does the risk of a patient representing with a cardiac adverse outcome affect your decision to admit patients to hospital as opposed to discharging them from the ED?
4) How often do you admit syncope patients in the 50 plus age group and why?
5) How much do you involve the patient in decision-making about your disposition decision?
    a. If so, under what circumstances?
    b. If not, why not?

6) Is syncope a medicolegal concern for you?
7) What do you think about the current resource utilisation, in general, in the work-up/management of syncope?
8) What is your opinion of the accelerated syncope strategy proposed in the ASPIRED study that enables a 14-day ECG patch to be placed on patients with unexplained syncope, in the ED?

9) How would this accelerated syncope strategy affect your patient disposal decision?
10) Tell me about any concerns you have about the accelerated syncope strategy proposed in the ASPIRED study.
11) Is there anything else you would like to tell me about the management of syncope in the ED?

That concludes our interview. Thank you again for your time.
