# Supplementary material for: Usability and acceptability of ambulatory monitoring in undiagnosed syncope: insights from the ASPIRED-Q qualitative study
Source: BMJ Open. 2025 Apr 8;15(4):e095927. doi: 10.1136/bmjopen-2024-095927 (PMC11979494; doi:10.1136/bmjopen-2024-095927)
Supplement: Supplementary file 4 [file bmjopen-2024-095927.reviewer_comments.pdf]

PEER REVIEW HISTORY

BMJ Open publishes all reviews undertaken for accepted manuscripts. Reviewers are asked to complete a checklist review form and are provided with free text boxes to elaborate on their assessment. These free text comments are reproduced below.

ARTICLE DETAILS

Title (Provisional)

The Usability and Acceptability of Ambulatory Monitoring in Undiagnosed Syncope: Insights from the ASPIRED-Q Qualitative Study

Authors

Pearsons, Alice; Hanson, Coral L; Neubeck, Lis; Blackstock, Caroline; Clarke, Ellise; Reed, Matthew James

---

VERSION 1 - REVIEW

**Reviewer**	**1**
**Name**	**Beasant, Lucy**
**Affiliation**	**University of Bristol, Centre for Academic Child Health**
**Date**	**13-Dec-2024**
**COI**	**None**

Thank you for submitting this interesting article to the BMJ Open for publication, I enjoyed reading it and it is very well written. I feel the manuscript requires some revisions prior to publication, please find detailed comments in the attached document.

Strengths and limitations of this study:

Page 4 of 80 - I think you particularly need to expand upon how the use of the 'theoretical framework of acceptability' enhanced the robustness of the findings to support this strength (see comments in results section below). Also, expand on the limitation: how challenges following up patient participants to interview after initial consent impacted on the study sample.

Introduction

Page 4 of 80 Line 12 *The majority of patient's (60%) had only experience one syncope interview*
Should this state episode?

Page 5 of 80 Lines 18-19 *Commonly used monitoring technologies include Holter monitors and Implantable Loop Recorders (ILR).* Can authors please provide a brief description of what 'standard care' was for control participants when discharged, and in the weeks following discharge. Did the 'intervention group' use the monitor purely for diagnosis of another episode over a 14 day period? A description of what participants received in each RCT arm could be provided in the Methods section or via a link/ref to already published work.

Page 5 of 80 Line 19 – '*access to these services is plagued by lengthy delays*.' Are references available to support this assertion/how long are the delays?

Page 6 of 80 line 8 states that the devices may '*lead to better adherence to monitoring protocols*' but it is unclear to the reader what 'monitoring protocols' patients in each study arm were following post discharge.

Methods

Page 8 of 80 line 15 – '*Data were analysed via thematic analysis*' (ref 15) The use of this reference at this point in the paper implies the 'reflexive thematic analysis' developed by Braun and Clarke was used, but then the authors *cite 'five stage framework approach to TA had been used*' (ref 16). This may cause confusion for readers, either remove Braun/Clarke reference or describe how this was applied to the TA process alongside the framework approach to TA.

Page 8 of 80 lines 25-26 It's unclear how the seven initial themes (experience of syncope, acute care journey, acceptability and usability of the intervention, decision making, communication, resources and impact on life) were refined, developed and resulted in the three overarching themes and seven subthemes.

Page 9 of 80 - lines 5-6 '*This process highlighted the need to apply a theoretical framework of acceptability to the developing themes*.' Please can the authors explain how/why the TA process (7 initial themes to 3 over-arching themes) highlighted the need to apply the theoretical framework of acceptability.

Results

Page 9 of 80 - line 22-23 – please provide average interview duration and range for each sub-sample (patients/HCPs).

Page 10 of 80 line 8 *'Efficacy'* I can see structurally how the theme/sub-themes map onto the model/theoretical constructs, but the 1 word theme headings are very generic. Could an initial summary be included at the beginning of each theme to describe what each 'main' theme is about as a whole?

Page 11 of 80 lines 4-25 Lots of points are made in relation to '*Patient Reassurance'* but few quotations are included in the table to support these findings – can more supporting quotation be added to the table?

Page 11 of 80 lines 15-18 Is the following point:

Despite this, one intervention group participant felt that wearing the device was the reason he continued to think about the possibility of repeat events, suggesting that without this they would have not had these ongoing concerns.

Supported by the following participant 12 quote in table 1 Page 13 of 80:

'And in a sense, it could be said that the only thing that had brought it into my mind that I might have this fainting fit again was the fact that the device that I was given was set up to deal with the fact that this might happen again. So, I think that brought the idea of the possibility of it happening again into my mind where it wouldn't have been there otherwise. (Participant 12, control group)

If so, this participant is identified as being in the control group, in which case I thought they would not be wearing the intervention monitor?

Page 12 of 80 Table 1 *Patient reassurance*, HCP 6 quote – add word [intervention] between words *helped* and *patients*

Page 14 of 80 line 10 add full stop: *diverse patient group.*

Page 14 of 80 Table 2 *Ease of use* (Participant 21) – add 'intervention group' as identifier for consistency - (Participant 21, intervention group)

Page 16 of 80 Explanation - *'This lack of involvement was further felt by a perceived lack of discharge advice'* (lines 5- 6) contradicts *'patients generally felt sufficiently verbally informed'* (line 12).

Page *16 of 80 lines 6-8 'This was exacerbated in the control group who did not benefit from the additional syncope advice experienced as part of the device instruction set up, which included a leaflet on syncope.' Seems* at odds with lines 12-13 '*no obvious differences in terms of acceptability were noted between the two groups.'*

Page 16 of 80 line 13-14 – *'however, patients highly valued receiving written information in the form of a leaflet'* – is the leaflet referring to the leaflet on syncope which only the intervention group got, or did control group get another leaflet? Or is this referring to the PIS and therefore relevant to both groups?

Page 16 of 80 line 19 Sub-theme heading '*Healthcare professional Training'* does not correspond with Table 3 sub-theme heading *'Clinician Training'* on next page.

Page 17 of 80 lines 1-2 '*Two healthcare professionals reported that receiving feedback from patients monitoring periods reinforced their decision-making processes*

and Table 3 quote from HCP 1 *'Because especially after I had the feedback …*

It would be useful to have a description of how the HCP got this feedback, so the process can be written into future training adequately (via routine ED follow up or study follow-up because patient took part in the RCT).

Page 18 of 80 lines 4-5 *Participants considered that the benefits of such a device were its capacity to improve clinical decision-making, and to reassure patients*. - should this state Health professionals *considered that the benefits*? and lines 8-10 *Participants considered that improved written information about device use and clinician training were necessary to ensure that the device could be successfully implemented in routine practice* - clarify that this is referring to patient data (written information?) and HCP data (clinician training) or just HCP.

Check throughout results section: when using 'participants' this refers to patients and health professional findings.

Page 18 of 80 line 14 – 'Previous systematic review 'should be 'A Previous systematic review'

Page 19 of 80 Lines 23-24 *The use of the Theoretical Framework of Acceptability provided a structured analysis of themes enhancing the robustness of the findings*. How did this improve the robustness of findings?

Page 29 of 80 COREQ checklist & Page 38 of 80 Protocol

Data saturation is mentioned in the protocol, but the concept was not utilised (COREQ checklist item 22) Can the authors explain why they chose not to include this and how they determined they had collected sufficient data?

Data will be analysed iteratively and the final participant number based on data

saturation which, for this study will be defined as no new themes being identified over

interviews and, in the opinion of the researcher, being unlikely to occur.

Page 25 of 80 Staff interview questions

Question 5 - How much do you involve the patient in decision-making about your disposition decision? a. If so, under what circumstances? b. If not, why not?

Considering that this paper reports patient feedback about a lack of involvement in decision-making, did HCPs expand upon why this happens / how patients might be more involved in future if this is appropriate?

Page 26 of 80 Patient interview questions

Question 9 probing questions - diagram or graphic about health risk be useful?

This diagram is not discussed in the paper – are these findings reported elsewhere? It might be useful to know if patients said they would find this useful?

Reviewer 2

Name Lampert, Rachel

Affiliation Yale Univ

Date 30-Dec-2024

COI	None

In this manuscript, Pearson et al describe results of a qualitative study investigating the usability of a patch monitor for syncope patients on discharge from the ED, in a subset of patients enrolled in the ASPIRED RCT of use of noninvasive monitors initiated during the index ED visit, and clinicians caring for them. They report that both groups expressed positive perspectives around efficacy and burden with clinicians emphasizing the need for education and structured pathways. The most effective and efficient way to diagnose syncope is not yet determined, as testing is often normal and etiology often not determined despite costly admissions and testing protocols. Thus the parent trial, has high potential clinical relevance. The current qualitative substudy provides helpful ancillary information. Methods are appropriate. There are aspects of the results and discussion that should be clarified as below but overall the study will support the clinical goal of improving syncope evaluation efficacy and efficiency.

Specific comments:

The device is described as "novel" but noninvasive patch monitors which are water-resistant and have no wires, are widely used and standard of care.

It would be helpful to provide, briefly, further details of the parent trial—what are the endpoints, the expected number?

While the labels on the themes are helpful to the investigators in categorizing, they are not clear to the reader. Eg, does "improved clinical care pathway" mean that the intervention arm led to this? Or that this is in general a need in syncope evaluation?

Opinions expressed by HCPs and patients are sometimes difficult to distinguish and should be separated and identified more clearly. Would avoid general summaries of "participants" opinions and separate into what patients and what HCP's expressed.

Overall, it is difficult to determine whether the clinicians are hypothesizing, or are basing their answers on their experience with the intervention/device.

Specific eg, P 9, line 14: It is unclear whether the ED clinicians, are involved in the final clinical diagnosis, and similarly, are they hypothesizing that clinical decision-making will be enhanced, or based on their experience in these patients, that the intervention did in fact enhance clinical decision-making?

Similarly, under "integration with current workflow" opinions are reported as "would be…", "could be.." If these clinicians were part of the study what was their actual experience?

P 10, line 11—it is noted that the control group expressed residual fear. Did the intervention group? While qualitative studies cannot provide comparisons, it can be stated whether anyone in the intervention group expressed this.

P 10 line 24—it should be clarified that this was a statement from a HCP and not a patient.

P 18 line 5 raises a crucial point in general, re who will follow up with the results. It would be helpful to explain enough about the parent study intervention to understand this aspect.

VERSION 1 - AUTHOR RESPONSE

Reviewer Comments	Author response
Reviewer 1	
The device is described as "novel" but noninvasive patch monitors which are water-resistant and have no wires, are widely used and standard of care. It would be helpful to provide, briefly, further details of the parent trial—what are the endpoints, the expected number?	We have now added this information in the introduction along with a reference to the study protocol. We have also included additional information based on comment from reviewer 2 which can also be found in the introduction.<br><br>The new text is:<br>"Whilst non-invasive ambulatory ECG monitors are indeed widely used by some cardiology teams, the ASPIRED trial is the first large study to look at applying a device immediately after the presenting syncope episode (i.e. from the ED) and monitoring for an enhanced period (i.e. 2 weeks). The study will recruit 2234 adults 16 years or older presenting with syncope remaining unexplained after initial assessment with a primary objective to determine whether the intervention decreases the number of self-reported episodes of syncope at one year compared to standard care monitoring."
While the labels on the themes are helpful to the investigators in categorizing, they are not clear to the reader. Eg, does "improved clinical care pathway" mean that the intervention arm led to this? Or that this is in general a need in syncope evaluation?	Reviewer 2 requested that all main themes be accompanied by a sentence or two of description to better frame what we were trying to convey. We believe that this has helped address your concern here.

Opinions expressed by HCPs and patients are sometimes difficult to distinguish and should be separated and identified more clearly. Would avoid general summaries of “participants”	We have now gone through the results and where participant(s) has been used we have expanded the term to either “HCP

opinions and separate into what patients and what HCP's expressed.	participant(s); patient participant(s); or HCP and patient participant(s) to aid clarity.
Overall, it is difficult to determine whether the clinicians are hypothesizing, or are basing their answers on their experience with the intervention/device.<br>Specific eg, P 9, line 14: It is unclear whether the ED clinicians, are involved in the final clinical diagnosis, and similarly, are they hypothesizing that clinical decision-making will be enhanced, or based on their experience in these patients, that the intervention did in fact enhance clinical decision-making?<br>Similarly, under "integration with current workflow" opinions are reported as "would be…", "could be.." If these clinicians were part of the study what was their actual experience?	Thank you for highlighting this. This is a little ambiguous because although those recruited were aware of the trial and helped recruit, they themselves were not involved in the device attachment or follow up as this is managed by the trial research teams. To address this, we have made this clearer in the 'methods-participants' section by clearly describing the involvement.<br><br>"Healthcare professionals recruited were aware of the trial and device and may have helped identify patients for the main recruitment. However, they were not involved in the application or delivery of the intervention which was managed via research teams at each site."
P 10, line 11—it is noted that the control group expressed residual fear. Did the intervention group? While qualitative studies cannot provide comparisons, it can be stated whether anyone in the intervention group expressed this.	We have now amended the following sentence to this comment to make it clearer that the intervention helped with this, but like you mentioned it is not possible to make an exact comparison<br><br>"This fear was not as pronounced in the intervention group as there was a feeling that they would receive 'quicker' help than waiting for a GP."
P 10 line 24—it should be clarified that this was a statement from a HCP and not a patient.	By going through the results and clarifying the HCP and patient participants we feel this has also helped to clarify this point.
P 18 line 5 raises a crucial point in general, re who will follow up with the results. It would be helpful to explain enough about the	This information has now been included in the 3rd paragraph of the discussion:

parent study intervention to understand this aspect.	"In ASPIRED, 14-day ambulatory ECG results were shared with the participant's responsible treating clinicians to action (usually the inpatient speciality physician and any relevant outpatient physicians responsible for ongoing care). ECG results were also placed in the participant's health record to be available to future treating clinician."
Reviewer 2	
Page 4 of 80 - I think you particularly need to expand upon how the use of the 'theoretical framework of acceptability' enhanced the robustness of the findings to support this strength (see comments in results section below).	Thank you it is really nice to be able to expand this section, the methods now reads:<br><br>"By doing so, it is possible to assess the anticipated and experienced acceptability of the intervention grounded in theory. "The seven-component framework includes affective attitude, burden, perceived effectiveness, ethicality, intervention coherence, opportunity costs and self-efficacy (20). Therefore, the model was used to increase robustness of analysis by applying the initial themes against predefined components for acceptability, which increased coherence and consistency of interpretation within the systematic framework. This helped refine the initial themes into main and subthemes to aid understanding."
Also, expand on the limitation: how challenges following up patient participants to interview after initial consent impacted on the study sample.	We have now expanded this point to say:<br><br>"This may have introduced bias by perhaps interviewing patients that were more satisfied with their care, or had more positive experiences, therefore those who had negative experiences or felt the intervention was ineffective might have been underrepresented."

Page 4 of 80 Line 12 *The majority of patient's (60%) had only experience one syncope Interview* Should this state episode?	As they are involved in the trial this was written to highlight that at the time they were interviewed they had only had one syncope episode. To avoid confusion, we have removed reference to interview and amended to:<br>"and 60% (n=12) experienced only a single syncope episode."
Page 5 of 80 Lines 18-19 *Commonly used monitoring technologies include Holter monitors and Implantable Loop Recorders (ILR).*<br>*a)* Can authors please provide a brief description of what 'standard care' was for control participants when discharged, and in the weeks following discharge. Did the 'intervention group' use the monitor purely for diagnosis of another episode over a 14-day period? A description of what participants received in each RCT arm could be provided in the Methods section or via a link/ref to already published work.	The original manuscript contains the link to the ISRCTN registration for the ASPIRED RCT however we have now also included additional information and a link to the published protocol. To make it clearer what is involved in the study we have added additional text into the introduction (see below) and have now included the schematic diagram do the RCT as a supplementary file 4.<br><br>"The control arm involved patients receiving all care usually given to unexplained syncope patients at each participating site along with some form of standard local monitoring such as but not limited to wired inpatient telemetry, Holter style monitoring or implantable loop recorder (Supplementary file 4)."
Page 5 of 80 Line 19 – *'access to these services is plagued by lengthy delays.'* Are references available to support this assertion/how long are the delays?	To make this more accurate we have now referenced the range in weeks based on several UK freedom of information requests to health boards for this data.<br><br>"However, access to these services is subject to delays. The average waiting time to receive a holter monitor ranges between 5-12 weeks [References available in manuscript]"
Page 6 of 80 line 8 states that the devices may *'lead to better adherence to monitoring protocols'* but it is unclear to the reader what 'monitoring protocols' patients in each study arm were following post discharge	We have now clarified that this is in relation to wearing the monitor which for holter monitors is uncomfortable. The reference supplied provides evidence for this. The new text reads:

	"These devices are designed to improve patient comfort and compliance due to their user-friendly features, which may lead to better patient adherence to continued wearing of the monitoring and subsequently higher diagnostic yields"
Methods:	
Page 8 of 80 line 15 – '*Data were analysed via thematic analysis'* (ref 15) The use of this reference at this point in the paper implies the 'reflexive thematic analysis' developed by Braun and Clarke was used, but then the authors *cite 'five stage framework approach to TA had been used*' (ref 16). This may cause confusion for readers, either remove Braun/Clarke reference or describe how this was applied to the TA process alongside the framework approach to TA.	Thank you. We were referencing Braun and Clark as the prominent researchers/literature on thematic analysis. However, we did use the framework approach so as advised we have removed the Braun and Clark reference to avoid confusion and updated the reference to:<br><br>Gale, N. K., et al. (2013). "Using the framework method for the analysis of qualitative data in multi-disciplinary health research." BMC Medical Research Methodology **13**(1): 117.
Page 8 of 80 lines 25-26 It's unclear how the seven initial themes (experience of syncope, acute care journey, acceptability and usability of the intervention, decision making, communication, resources and impact on life) were refined, developed and resulted in the three overarching themes and seven subthemes.	We have revised the manuscript to provide a more detailed explanation of this process (significant changes please see Methods-data analysis). We also feel this has now been addressed by the clarification requested regarding additional information regarding how the use of the theoretical model increases robustness.
Page 9 of 80 - lines 5-6 *'This process highlighted the need to apply a theoretical framework of acceptability to the developing themes.'* Please can the authors explain how/why the TA process (7 initial themes to 3 over-arching themes) highlighted the need to apply the theoretical framework of acceptability.	We have now added further explanation:<br><br>"Specifically, the initial themes highlighted a complex interaction between patients' understanding of the intervention, its perceived usability, and the broader context of their medical experiences. As we refined the analysis, it became evident that these experiences were multifaceted and not just related to practical concerns (such as the usability of the intervention) but were also

	deeply intertwined with what participants deemed acceptable or unacceptable in terms of their treatment and decision-making processes.”
Results	
Page 9 of 80 - line 22-23 – please provide average interview duration and range for each sub-sample (patients/HCPs).	Amended:<br>“Interviews ranged from 8 minutes to 36 minutes (patients) and 36 minutes to 57 minutes (HCPs).”
Page 10 of 80 line 8 *‘Efficacy’* I can see structurally how the theme/sub-themes map onto the model/theoretical constructs, but the 1 word theme headings are very generic. Could an initial summary be included at the beginning of each theme to describe what each ‘main’ theme is about as a whole?	We have now added an explanatory statement for each main theme. The subthemes are defined within the main body of results text. The descriptions read:<br><br>Efficacy: “The theme of efficacy reflects patient and HCP participant perceptions of how effective the intervention was in improving syncope management, influencing confidence in the intervention, and ultimately the overall experience and acceptability of the intervention.”<br><br>Burden: “The theme of burden patient and HCP participant perceptions of the physical, emotional, and logistical challenges associated with the intervention.”<br><br>Communication and Education: “The theme of communication reflects patient and HCP participant perceptions of the clarity and effectiveness of information provided by healthcare professionals which directly influenced patient sense of acceptability and engagement.”
Page 11 of 80 lines 4-25 Lots of points are made in relation to ‘*Patient Reassurance’* but few quotations are included in the table to support these findings – can more supporting quotation be added to the table?	We feel that we have already included a reasonable number of quotes here (4) - 2 from HCPs and 2 from patients. Patient reassurance is discussed a little more than the other subthemes as as unlike the other two subthemes, this includes data from

	both HCPs and Patients so importantly brings the perspective of both groups into the discussion.
Page 11 of 80 lines 15-18 Is the following point:<br>*Despite this, one intervention group participant felt that wearing the device was the reason he continued to think about the possibility of repeat events, suggesting that without this they would have not had these ongoing concerns.* Supported by the following participant 12 quote in table 1 Page 13 of 80: *'And in a sense, it could be said that the only thing that had brought it into my mind that I might have this fainting fit again was the fact that the device that I was given was set up to deal with the fact that this might happen again. So, I think that brought the idea of the possibility of it happening again into my mind where it wouldn't have been there otherwise. (Participant 12, control group)* If so, this participant is identified as being in the control group, in which case I thought they would not be wearing the intervention monitor?	Thank you very much for picking this up. You are absolutely correct, and we have now amended Participant 12 to the intervention group.
Page 12 of 80 Table 1 *Patient reassurance*, HCP 6 quote – add word [intervention] between words *helped* and *patients*	Amended
Page 14 of 80 line 10 add full stop: *diverse patient group.*	Thank you
Page 14 of 80 Table 2 *Ease of use* (Participant 21) – add 'intervention group' as identifier for consistency - (Participant 21, intervention group)	Thank you
Page *16 of 80 lines 6-8 'This was exacerbated in the control group who did not benefit from the additional syncope advice experienced as part of the device instruction set up, which included a leaflet on syncope.' Seems* at odds with lines 12-13 '*no obvious differences in terms of acceptability were noted between the two groups.'*	The difference between two groups refers to whether the intervention group received advice in person versus over the phone rather than between the intervention and control group. We have therefore re-written this to avoid further confusion. It now reads:

	"Regardless of the mode of communication, <u>intervention</u> participants generally felt sufficiently verbally informed, and no obvious differences in terms of acceptability were noted between the <u>methods of information delivery</u>."
Page 16 of 80 line 13-14 – *'however, patients highly valued receiving written information in the form of a leaflet'* – is the leaflet referring to the leaflet on syncope which only the intervention group got, or did control group get another leaflet? Or is this referring to the PIS and therefore relevant to both groups?	Thank you- this is now clarified, and we believe now reads better when joined with your above comment. It now reads:<br><br>"However, written information in the form of a leaflet provided with the device was highly valued."
Page 16 of 80 line 19 Sub-theme heading '*Healthcare professional Training'* does not correspond with Table 3 sub-theme heading '*Clinician Training'* on next page.	Thank you we have now amended this to "healthcare professional training" which now also matches the diagram. We have also made sure than clinician has not been used throughout the manuscript changing all reference to healthcare professional for consistency.
Page 17 of 80 lines 1-2 '*Two healthcare professionals reported that receiving feedback from patients monitoring periods reinforced their decision-making processes* and Table 3 quote from HCP 1 *'Because especially after I had the feedback …*<br>It would be useful to have a description of how the HCP got this feedback, so the process can be written into future training adequately (via routine ED follow up or study follow-up because patient took part in the RCT).	We agree this would have been helpful, unfortunately this information is not available.
Page 18 of 80 lines 4-5 *Participants considered that the benefits of such a device were its capacity to improve clinical decision-making, and to reassure patients.* - should this state Health professionals *considered that the benefits*?<br><br>and lines 8-10 *Participants considered that improved written information about device use and clinician training were necessary to ensure that the device could be successfully*	This has been amended as part of reviewer 1s point regarding better delineating between patients and HCPs. It now reads:<br><br>"HCP and patient participants considered that the benefits of such a device were its capacity to improve clinical decision-making, and to reassure patients. The device was considered easy to use by patient participants, despite some reported technical issues. HCP participants reported that it could be

implemented in routine practice - clarify that this is referring to patient data (written information?) and HCP data (clinician training) or just HCP.	integrated into their workflow but that there was a need for a defined pathway to ensure appropriate use. HCP and patient participants considered that improved written information about device use and clinician training were necessary to ensure that the device could be successfully implemented in routine practice."
Check throughout results section: when using 'participants' this refers to patients and health professional findings.	This has been amended as above and are grateful that both reviewers highlighted this as confusing so that we could improve readability and understanding.
Page 18 of 80 line 14 – 'Previous systematic review 'should be 'A Previous systematic review'	Amended
Page 19 of 80 Lines 23-24 *The use of the Theoretical Framework of Acceptability provided a structured analysis of themes enhancing the robustness of the findings*. How did this improve the robustness of findings?	We have now expanded on this significantly in the results and believe that this sentence now stands alone due to this additional information earlier in the manuscript.
Page 29 of 80 COREQ checklist & Page 38 of 80 Protocol Data saturation is mentioned in the protocol, but the concept was not utilised (COREQ checklist item 22) Can the authors explain why they chose not to include this and how they determined they had collected sufficient data?<br>*Data will be analysed iteratively and the final participant number based on data saturation which, for this study will be defined as no new themes being identified over interviews and, in the opinion of the researcher, being unlikely to occur.*	We have now discussed this in limitation. Recruitment challenges of HCPs were not mentioned, and this is where data saturation was not reached due to recruitment challenges. Therefore, the following has now been added to the limitation section:<br><br>"It is unlikely that data saturation was reached within the HCP participant group. This was due to challenges engaging this group, in particular recruiting nursing professionals e.g. emergency nurse practitioners. It is hypothesised by the authors that this relates to the lack of admin time provided to this professional group. Therefore, the findings of the healthcare professionals should be interpreted with caution as it may not be representative of the ED as a whole."

Page 25 of 80 Staff interview questions Question 5 - How much do you involve the patient in decision-making about your disposition decision? a. If so, under what circumstances? b. If not, why not? Considering that this paper reports patient feedback about a lack of involvement in decision making, did HCPs expand upon why this happens / how patients might be more involved in future if this is appropriate?	This was very much discussed by all HCPs and all believed they provided comprehensive discharge advice so we have now added in to this section the following:<br><br>"This lack of involvement was further felt by a perceived lack of discharge advice. This was contradicted by healthcare professionals where all HCP participants reported that they provided comprehensive discharge advice with a focus on red flag warnings and clearly communicating that they were unable to find a cause but could rule out anything serious."
Page 26 of 80 Patient interview questions Question 9 probing questions - diagram or graphic about health risk be useful? This diagram is not discussed in the paper – are these findings reported elsewhere? It might be useful to know if patients said they would find this useful?	A diagram was not used due to the interviews being conducted via telephone; this original question was designed for online interviews which were not undertaken due to ethical constraints. Therefore, conversation focused on describing what patients would have wanted. The outcome of this was discussed in the "communication and education" theme regarding what information patients received/would have liked. However, for the pragmatic application of this manuscript we only focused on device related information.

VERSION 2 - REVIEW

**Reviewer**	**2**
**Name**	**Lampert, Rachel**
**Affiliation**	**Yale Univ**
**Date**	**23-Feb-2025**

COI

The authors have been highly responsive to the prior reviews with all noted details and clarifications now included
